# Effects of glycine and n-acetylcysteine on glutathione levels and mitochondrial energy metabolism in healthy aging

**DOI:** 10.1093/geroni/igab046.2574

**Published:** 2021-12-17

**Authors:** Philipp Gut, Giulia Lizzo, Eugenia Migliavacca, Leonidas Karagounis, Tim Heise, Maximilian Eynatten

**Affiliations:** 1 Cell Biology, Nestlé Institute of Health Sciences, Nestlé Research, Lausanne, Vaud, Switzerland; 2 Nestlé Research, Lausanne, Vaud, Switzerland; 3 Nestlé Health Science, Vers-chez-les Blanc, Vaud, Switzerland; 4 Profile Institut for Metabolic Research, Neuss, Baden-Wurttemberg, Germany

## Abstract

Glutathione is an intracellular antioxidant that neutralizes reactive oxygen species and prevents tissue damage. Dietary supplementation with the glutathione precursors glycine and n-acetylcysteine supports the maintenance of normal glutathione levels in several age-related diseases, but the optimal doses and their efficacy in healthy elderly are not established. We report results from a randomized controlled clinical trial in 114 healthy volunteers (mean age = 65 years) receiving glycine and n-acetylcysteine (GlyNAC) at three different doses for two weeks (1.2g/1.2, 2.4g/2.4g, 3.6g/.3.6g of each amino acid). Older subjects showed increased oxidative damage and a lower reduced-to-oxidized glutathione ratio (GSH:GSSG) compared to young subjects, but unchanged total glutathione levels. GlyNAC did not increase levels of circulating glutathione compared to placebo treatment, the primary study endpoint. However, stratification analyses suggest that subjects with high oxidative stress and low glutathione status responded with glutathione generation. We find that unrelated to glutathione status, healthy aging was associated with lower levels of fasting glycine that can be increased towards those observed in young subjects with supplementation. Using preclinical models, we find that tissue glycine depletion is a common feature of healthy aging. Supplementation of old mice with glycine efficiently improved age-related decline of mitochondrial respiratory function in skeletal muscle and prevented a gene program associated with protein catabolism observed in control-treated animals. In conclusion, GlyNAC is safe and well-tolerated and may selectively increase glutathione levels in older subjects with oxidative stress and glutathione demand. Our data further suggest that glycine may support mitochondrial function independently of NAC.

